# In Vitro Evaluation of the Effects of Commercial Prebiotic GOS and FOS Products on Human Colonic Caco–2 Cells

**DOI:** 10.3390/nu12051281

**Published:** 2020-04-30

**Authors:** Geraldine M. Flaujac Lafontaine, Neville M. Fish, Ian F. Connerton

**Affiliations:** 1 Division of Microbiology, Brewing and Biotechnology, School of Biosciences, University of Nottingham, Sutton Bonington Campus, Loughborough LE12 5RD, UK; geraldine.lafontaine@nottingham.ac.uk; 2 Saputo Dairy UK, Innovation Centre, Harper Adams University, Newport TF10 8NB, UK; nmfish@gmail.com

**Keywords:** prebiotics, oligosaccharides, GOS, FOS, RNA-seq, transcriptome, functional pathway analysis, Caco–2, polarized monolayers

## Abstract

Prebiotic oligosaccharides are widely used as human and animal feed additives for their beneficial effects on the gut microbiota. However, there are limited data to assess the direct effect of such functional foods on the transcriptome of intestinal epithelial cells. The purpose of this study is to describe the differential transcriptomes and cellular pathways of colonic cells directly exposed to galacto-oligosaccharides (GOS) and fructo-oligosaccharides (FOS). We have examined the differential gene expression of polarized Caco–2 cells treated with GOS or FOS products and their respective mock-treated cells using mRNA sequencing (RNA-seq). A total of 89 significant differentially expressed genes were identified between GOS and mock-treated groups. For FOS treatment, a reduced number of 12 significant genes were observed to be differentially expressed relative to the control group. KEGG and gene ontology functional analysis revealed that genes up-regulated in the presence of GOS were involved in digestion and absorption processes, fatty acids and steroids metabolism, potential antimicrobial proteins, energy-dependent and -independent transmembrane trafficking of solutes and amino acids. Using our data, we have established complementary non-prebiotic modes of action for these frequently used dietary fibers.

## 1. Introduction

Prebiotics are generally defined as substances that are selectively utilized by host microorganisms to produce a health benefit [1]. Prebiotic oligosaccharides conform to the definition as non-digestible food ingredients that beneficially affect the host by stimulating the growth and/or activity of beneficial bacteria in the colon [2]. Such oligosaccharides are widely used as human and animal nutritional additives for their beneficial effects on the composition of the probiotic microbiota and gut health [3,4]. However, oligosaccharides that largely resist hydrolytic activities of salivary and intestinal digestive enzymes reach the colon virtually intact, thus prompting to question whether they could have a direct or “non-prebiotic” effect on the intestinal epithelium should they reach the mucosa.

Galacto-oligosaccharides (GOS or β-GOS) are produced through the β-galactosidase catalyzed transgalactosylation of lactose creating oligosaccharides with degrees of polymerization (DP) ranging from 2 to 8 monomeric units with β(1→3), β(1→4) and β(1→6) linkages between galactose units and are usually coupled to a terminal glucose [5]. Commercial GOS products typically also contain residual lactose, glucose and galactose reactants. Fructo-oligosaccharides (FOS) are commonly produced by acid- or enzyme-catalyzed hydrolysis of inulin [6], which results in fructan oligomers of 1 to 7 units (DP 1–7), sometimes with a terminal glucose [6,7]. Commercial FOS products typically also contains residual sucrose, fructose and glucose.

In vivo, GOS supplementation has been shown to increase iron absorption from micronutrient powder in Kenyan infants [8] or to reduce stress-induced gastrointestinal dysfunction and days of cold or flu symptoms in controlled trials of healthy university students [9]. In a healthy volunteer study, Schmidt et al. [10] described the reduction of the salivary cortisol awakening response associated with GOS administration, implying suppression of the neuroendocrine stress response. However, Liu et al. [11] suggested short-term intake of high-dose GOS and FOS prebiotics had an adverse effect on glucose metabolism despite increased *Bifidobacterium* in the fecal microbiota. In controlled animal studies, GOS has been associated with improved growth performance of broiler chickens [12] or during exposure to heat stress [13,14]. Dietary supplementation of GOS has also been linked with improvements in the transition to a mature intestinal microbiota in broiler chickens [12,15] and in suckling piglets [16,17]. In a study conducted with suckling male rats, in addition to changes in the intestinal microbiota, Le Dréan et al. [18] observed GOS and FOS supplementation impacted entero-endocrine cell maturation by bringing about transient increases in the density of GLP-1 cells and the production of the satiety-related peptides GLP-1 and PYY.

The direct cellular effects of prebiotics have been investigated using polarized intestinal epithelial cell models. For example, GOS exposure has been associated with improved tight junction formation and re-epithelialization post disruption, which coincided with increases in the expression of cell proliferation and cell differentiation pathways [19]. GOS was also reported to prevent deoxynivalenol-induced compromise of the integrity of Caco–2 cell monolayers and the corresponding decline in the expression of the tight junction encoding gene CLDN3 [20]. GOS is also reported to reduce the adherence of *Salmonella* Typhimurium to mucus- and to non-mucus-producing HT–29 cells [21].

Several studies have reported that GOS, FOS and inulin promote calcium absorption in the animal and human gut [22,23,24]. FOS has also been reported to have non-microbiome or non-prebiotic mediated effects on immune function. For example, Fransen et al. used germ-free mice to confirm microbiota-independent changes in immune function with short- or long-chain β2→1-fructans enhancing T helper 1 cells in Peyer’s patches and short-chain β2→1-fructans, increasing regulatory T cells and CD11b−CD103− dendritic cells in the mesenteric lymph nodes [25]. FOS-inulin pre-incubation of a chicken macrophage cell line HD11 before challenge with *Salmonella* Enteriditis reduced cellular uptake of the pathogen and IL-1β gene expression, suggesting that inulin-enriched FOS had the ability to modulate the innate immune response [26].

Caco–2 cells are a continuous line of heterogeneous human epithelial colorectal adenocarcinoma cells that produce tight junctions, microvilli, enzymes and transporters characteristic of enterocytes. The Caco–2 monolayer is widely used in the pharmaceutical industry as an in vitro model of the human intestinal mucosa to predict the absorption of orally administered drugs. Caco–2 cells are commonly cultured as a polarized epithelial stratum by forming confluent monolayers on insert filters that provide a physical and biochemical barrier to the passage of ions and small molecules [27]. In this study, we have examined the effects of Nutrabiotic^®^ GOS and Beneo^®^ FOS (also referred to as “GOS” and “FOS” respectively) on the integrity of the monolayers by measuring trans-epithelial electrical resistance (TEER), and on differential gene expression relative to mock treated control using whole transcriptome sequencing technology. Our data establish complementary non-prebiotic modes of action for these frequently used dietary fibers.

## 2. Materials and Methods

### 2.1. Oligosaccharides

Galacto-oligosaccharides (Nutrabiotic^®^ GOS, 66% w/w syrup) were provided by Dairy Crest Ltd (Esher, Surrey, UK) and Fructo-oligosaccharides (FOS Orafti^®^L95, 75% w/w syrup) were obtained from BENEO–Orafti (Oreye, Belgium). The detailed composition of the oligosaccharide treatments applied are summarized in Table 1. The GOS treatment media contained 2% v/v of Nutrabiotic^®^ GOS, equivalent to 1.4% w/v of DP 2–7+ galacto-oligosaccharides. The FOS treatment media contained 2% v/v of Orafti^®^L95 FOS, equivalent to 2% w/v of DP 2–8 fructo-oligosaccharides. To account for the presence of mono- and digestible di-saccharides contained in Nutrabiotic^®^ GOS and Orafti^®^L95 FOS syrups used for treatment, we tailored the mock-treatments accordingly for GOS by incorporating galactose (0.03%), glucose (0.4%) and lactose (0.2%) in the GOS mock control, and by inclusion of fructose (0.06%), glucose (0.004%) and sucrose (0.04%) in the FOS mock control.

### 2.2. Cells and Experimental Design

The microbiota-independent effects of GOS and FOS oligosaccharides were assessed in vitro using human Caco–2 cells cultured as a confluent monolayer and exposed to treatment for 24 h. Each treatment was undertaken as three technical replications of three independent biological replicates represented by different cell passages. The human colon adenocarcinoma Caco–2 cell line was obtained from the American Type Tissue Collection (ATCC^®^ HTB–37™, passage 18; lot 62381028; LGC Standards, Teddington, UK,) and propagated according to established methods [28]. In brief, cells were cultured in Dulbecco’s modified Eagle medium (DMEM) and seeded at a density of 10^5^ cells onto 1.12 cm^2^ Transwell^®^ permeable support with a 0.4 μm tissue culture treated polyester membrane placed in a 12-well plate (3460; Costar, Kennebunk, ME, USA). Caco–2 cells were maintained in a humidified atmosphere of 95% air and 5% CO_2_ at 37 °C. Fresh culture medium containing DMEM (D6546, Sigma, Gillingham, UK), sterile Fetal Bovine Serum 10% v/v (F9665, Sigma), L-glutamine 2 mM (G7513, Sigma), MEM non-essential amino acid solution supplement 1x (M7145, Sigma), penicillin 100 U/mL and streptomycin 100 µg/mL (P0781, Sigma) was replenished every 2 to 3 days. Control mono- and di-saccharides-containing “GOS mock” and “FOS mock” treatment media were prepared as indicated in Table 2. GOS and FOS treatment media were prepared by dissolving 2% (v/v) of the oligo-saccharides syrups in FBS-free and antibiotic-free cell culture DMEM. All treatment culture media were free from serum and antibiotics to eliminate any interference from extraneous molecules, proteins or hormones.

After 18 days of culture, a confluent monolayer was obtained with a TEER exceeding 400 Ωcm^2^ as measured by an EVOM voltohmmeter (World Precision Instruments, Sarasota, FL, USA). Independently, replicates of cells from passages ranging 22 to 26 were gently washed 3 times (at hourly intervals) with warm non-supplemented (serum-free, antibiotic-free) DMEM culture medium and finally replenished from apical and basolateral sides of the Transwell^®^ inserts with oligosaccharides or mock treatment media for 24 h exposure before being harvested for total RNA extraction.

### 2.3. Integrity of Tight Junctions

Development of tight junction formation was monitored at regular intervals during the monolayers expansion by measuring the TEER across each of the growth inserts with an EVOM voltohmmeter (World Precision Instruments) connected to a pair of chopstick electrodes. Ohmic resistance of a blank (culture insert with respective medium but without cells) was measured in parallel. To obtain the sample resistance, the blank value was subtracted from the total resistance of the sample-containing cells. Final unit area resistance (Ωcm^2^) was calculated by multiplying the sample resistance by the effective area of the membrane (1.12 cm^2^ for 12-wells Transwell^®^ inserts). To assess the effect of oligosaccharides on the Caco–2 cell monolayers, the electrical resistance across each cell monolayer was measured prior to exposure (T_0_) and after 24 h of exposure (T_24h_) to the treatment media.

### 2.4. RNA Extraction and Qualification

Cells were gently washed in situ twice with ice cold sterile PBS followed by addition of 500 µl of TRIzol™ reagent (ThermoFisher Scientific, Paisley, UK) ready for harvesting by directly scraping the cells from the culture surface. Harvested cells were transferred to a 2 mL Eppendorf^®^ safe-lock microcentrifuge tube for total RNA extraction according to TRIzol™ reagent manufacturer’s protocol. Purified RNAs were quality-assessed using Agilent TapeStation 2200 system (G2964AA; Agilent, Stockport, UK) with RNA ScreenTape Assay reagents (5067–5576; 5067–5577; 5067–5578 Agilent). The RNA preparations had RIN values within the range 8.2 to 9.7 (mean = 9.24), which were subsequently aliquoted to limit freeze-thaw cycles and stored in −80 °C until cDNA library preparation.

### 2.5. Library Preparation for mRNA Sequencing

RNA concentrations were measured using Qubit Fluorometer and Qubit RNA BR Assay Kit (Q10211; ThermoFisher Scientific). A Biomek 4000 Automated Workstation (Beckman Coulter, High Wycombe, UK) was used to prepare sequencing libraries. mRNA was purified from 1 µg of total RNA using the NEBNext Poly(A) mRNA Magnetic Isolation Module (E7490; New England Biolabs, Ipswich, UK). Indexed sequencing libraries were then prepared using the NEBNext Ultra directional RNA Library Preparation Kit for Illumina (E7760; New England) and NEBNext Multiplex Oligos for Illumina, Index Primers Sets 1 and 2 (E7335 and E7500; New England Biolabs). Libraries were quantified using Qubit Fluorometer and Qubit dsDNA HS Kit (Q32854; ThermoFisher Scientific). Library fragment-length distributions were analyzed using the Agilent TapeStation 4200 with the Agilent High Sensitivity D1000 ScreenTape Assay (5067–5584 and 5067–5585; Agilent). Libraries were pooled in equimolar amounts and final library quantification performed using KAPA Library Quantification Kit for Illumina (KK4824; Roche, Pleasanton, CA, USA). The library pool was sequenced on an Illlumina NextSeq500 over two High Output 150 cycle kits (FC-404-2002; Illumina, San Diego, CA, USA), to generate over 40 million pairs of 75-bp paired-end reads per sample. The metadata and DNA sequences are available under the NCBI GEO series accession number GSE145303.

### 2.6. Quality Control and Mapping Analyses

High-throughput sequence reads were quality checked with FastQC (version 0.11.3). Raw reads in FASTQ format were subsequently processed using the Trim Sequences Module in CLC Genomics Workbench 12.03 (Qiagen, Aarhus, Denmark). In this step, clean data were obtained by removing reads from the raw data that contained adapter sequences and those of low-quality, all downstream analyses were performed with high-quality clean data. Using the RNA-Seq Analysis Module in CLC Genomics Workbench 12.03, clean trimmed reads were paired and mapped to *Homo sapiens* Hg38 (GRCh38 reference genome available in ENSEMBL) thus generating normalized counts of gene and transcript hits as RPKM (reads per kilobase of exon model per million mapped reads) and TPM (transcripts per million). Normalized counts were treated to compute P-values and fold changes (FC) for downstream differential expression analysis. Using the RNA-Seq Analysis Module from CLC Genomics Workbench 12.03, differential expression analysis between oligosaccharides-treated group and mock-treated group was determined using a negative binomial distribution model with the resulting P-values being adjusted to *p*-adj values using the approach of Benjamini and Hochberg [29] for controlling the false discovery rate (FDR). Genes with absolute FC ≥ 1.5 and *p*–adj value < 0.05 were designated as significantly differentially expressed (DE). Volcano plots of genes that were differentially expressed were developed using CLC workbench 12.03. Principal Component Analysis (PCA) was carried out using the web-based tool ClustVis [30]. Heatmap diagrams were developed using the Morpheus web-based tool [31] where rows and columns were clustered using Pearson’s correlation distance and average linkage. Venn diagram showing the overlap of differentially expressed genes between GOS and FOS treatments were calculated using the web-based tool InteractiVenn [32].

### 2.7. Functional Analysis of Differentially Expressed Genes

Functional analysis of significant differentially expressed genes was achieved by implementing bioinformatics tools on gene set enrichment and pathway databases analysis based on Gene Ontology (GO) [33,34] terms and the Kyoto Encyclopedia of Genes and Genomes (KEGG) [35] collection of databases. We investigated statistically significant gene sets modulated by GOS and FOS with | FC value | ≥ 1.5 and FDR *p*–adj. < 0.05 by implementing hypergeometric distribution within NIPA.Enrichment.R script (v0.6.7.R, https://github.com/ADAC-UoN/NIPA) and applying a minimum of 2 genes enriched, a cut-off *q*–value = 0.05 for KEGG and GO bases and an enrichment analysis established on “hsa” (*Homo sapiens*) Ensembl database. Briefly, NIPA.Enrichment.R script executed the bioconductor biomaRt [36,37] package to integrate Ensembl sequence data with data analysis using the GO tool, the Generally Applicable Gene set Enrichment for pathway (GAGE) package performed gene set analysis [38] and Pathview completed pathway-based data integration and visualization [39].

### 2.8. Quantitative Real-Time PCR Validation

Relative gene expression of DE genes computed by RNA-seq was established by RT-qPCR according to the Minimum Information for publication of Quantitative real-time PCR Experiments (MIQE) guidelines [40]. Sequences of RT-qPCR primers (Eurofins Genomics, Ebersberg, Germany) were designed using National Center for Biotechnology Information (NCBI) primer designing tool (http://www.ncbi.nlm.nih.gov/tools/primer-blast/) [41] and are described in Table 3. cDNA was synthesized using Invitrogen™ SuperScript™ II (18064014, ThermoFisher Scientific) according to the random hexamers primer method previously described [42]. Quantitative RT-PCR was performed using the Applied Biosystems PowerUP™ SYBR™ Green PCR Master Mix (A25742, ThermoFisher Scientific) on a Real-Time PCR LightCycler^®^ 480 System (Roche, Pleasanton, CA, USA). Each sample was processed in triplicate and the expression of genes of interest was normalized to endogenous housekeeping genes GAPDH, PUM1 and ACTB. Amplification protocol comprised one denaturation cycle at 95 °C for 5 min (ramp rate 4.8 °C/s), forty amplification cycles at 95 °C for 15 s, 65 °C for 30 s and 72 °C for 30s (ramp rate 1.5 °C/s). Melting curves were assessed for each RT-qPCR reaction using LightCycler^®^ 480 System software (v 1.5.1.62; Roche, Pleasanton). Resulting PCR product size was confirmed using Agilent TapeStation 2200 system (G2964AA; Agilent) with DNA D1000 ScreenTape Assay (5067-5582 and 5067-5583; Agilent). Primer sets were validated for specificity when melting curves analysis exhibited a single peak with Tm > 78 °C and amplified product size matched expected product size as established by the NCBI primer designing tool. Relative gene expression was calculated using the relative quantification method with amplification efficiency corrected calculation models [43,44]. Amplification rate and relative concentration was calculated using LightCycler^®^ 480 System software (v 1.5.1.62; Roche, Pleasanton) based on a linear regression slope established by 2-fold dilution series of a pool of all RNA samples.

### 2.9. Statistical Analysis

TEER experimental results are expressed as mean ± standard deviation, the differences between groups were evaluated by one-way ANOVA using Genstat 19th edition (VSNI Ltd, Hemel Hempstead, UK), where *p*-values < 0.05 were considered statistically significant. Within CLC Genomics Workbench 12.03, differential gene expression statistical analysis was based on Baggerly’s beta-binomial model [45] to account for between-library variability; two-sided *p*-values for the test are described as “*p*-value”. *p*-values were subsequently corrected for false discovery rate FDR (“*p*-adj values”) using the Benjamini and Hochberg’s approach [29]; *p*-adj value < 0.05 was considered significant. For functional analysis, a hypergeometric distribution model generated q-values [46]; a minimum of 2 genes enriched with q-values < 0.05 for KEGG and GO enrichment were used as the criteria for reporting.

### 2.10. Data Availability

The data discussed in this publication have been deposited in NCBI’s Gene Expression Omnibus database and are accessible through GEO series accession number GSE145303 (https://www.ncbi.nlm.nih.gov/geo/query/acc.cgi?acc=GSE145303). The project appears in the NCBI database within Bioproject PRJNA606703 (https://www.ncbi.nlm.nih.gov/bioproject/PRJNA606703).

## 3. Results

### 3.1. Influence of GOS and FOS on Epithelial Monolayer Tight Junction Integrity

**Figure 1:**
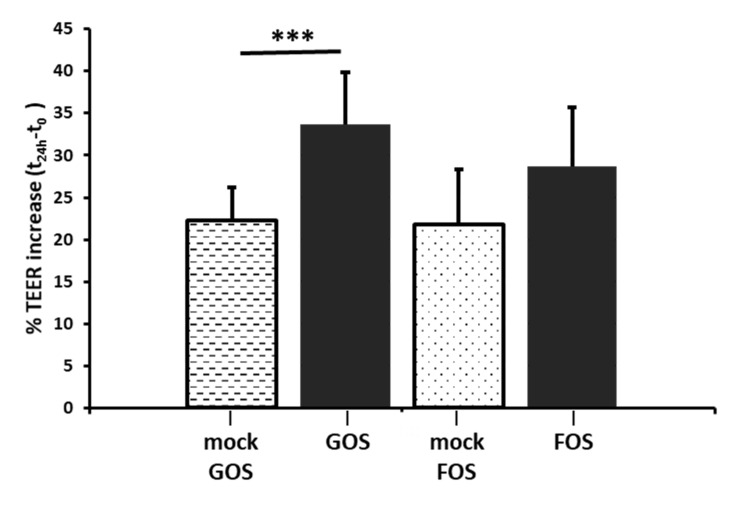
GOS and FOS effect on trans-epithelial resistance of Caco–2 monolayers. Polarized confluent Caco–2 monolayers were exposed for 24 h to GOS, FOS (2% v/v) or their respective mock media. GOS increased significantly the monolayer TEER (+33.62%, *p* < 0.001), while the TEER increase induced by FOS exposure was not statistically significant (+28.68%, *p* = 0.054). Data are expressed as means ± SD and were tested using ANOVA t-test, ***p < 0.001 (*n* = 3).

To assess the direct effect of dietary GOS and FOS, cells were exposed to tailored media containing the oligosaccharides. The presence of mono- and di-saccharides contained in GOS and FOS syrups used for treatment was accounted for in the control mock media for Nutrabiotic^®^ GOS 64% by incorporating galactose, glucose and lactose and by inclusion of fructose, glucose and sucrose for Orafti^®^L95 FOS. The treatment of Caco–2 cell monolayers with GOS generated a significant increase in the TEER values (Figure 1, TEER +33.62%, *p* = 0.00037) when compared to the mock-exposed cells. Similarly, FOS also induced greater TEER when compared to the mock-exposed cells (Figure 1, TEER +28.68%, *p* = 0.054). The rise in trans-epithelial electrical resistance of the monolayer suggest an acceleration of the tight junction dynamics that indicates an improvement of the monolayer integrity under the influence of the oligosaccharide treatments.

### 3.2. Transcriptome Sequencing and Functional Annotation

The effect of GOS and FOS preparations on the global transcriptome of Caco–2 cell monolayers were assessed by RNA-seq using the Illumina NextSeq sequencing platform. RNAs were harvested from three biological replicates for each treatment group and mRNA sequencing generated between 44 and 134 million paired-end reads of 75-bp per sample. An average of 88.67% good-quality paired-end reads per sample were mapped to Homo sapiens Hg38 reference genome. The summary of RNA-seq data and mapping for each sample are presented in Table 4. Gene and transcript normalized hit counts were used to generate FC and FDR adjusted *p*-values to implement differential gene expression analysis.

### 3.3. Analysis of Differential Gene Expression

**Figure 2:**
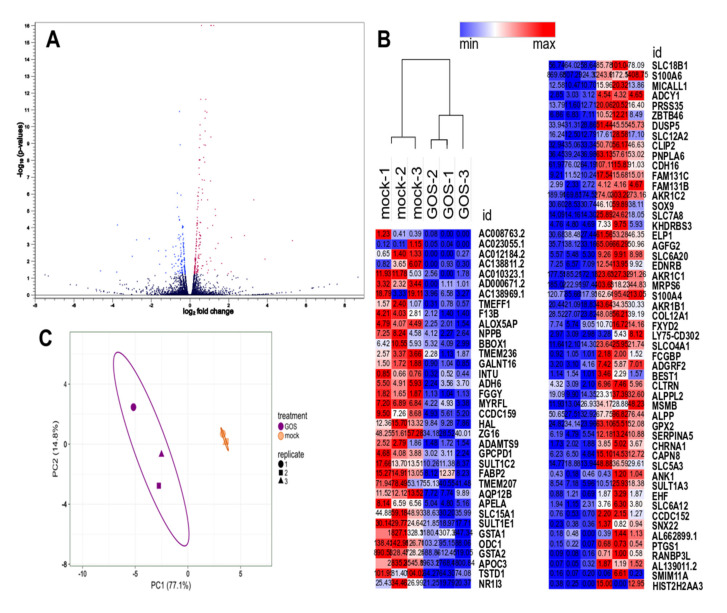
Differentially expressed genes associated with GOS treatment. (**A**) Volcano plot of genes differentially expressed between GOS and GOS mock treatment (*n* = 3). The abscissa represents log_2_ fold changes in gene expression, the ordinate represents statistical significance of the variations in gene expression, expressed as -log_10_(*p*-values). (**B**) Principal component scatter plot showing variance of the biological replicates for each treatment (*n* = 3). Axis x and y show principal components 1 (PC1) and 2 (PC2) that explain 77.1% and 14.8% of the total variance respectively. Prediction ellipses indicate probability 0.95 that a new observation from the same group will fall inside the ellipse; N = 6 data points. (**C**). Heatmap displaying differentially expressed genes for GOS and mock treatments. Rows and columns are clustered using Pearson’s correlation distance and average linkage. Data are presented as normalized transcripts count (TPM) with |FC| ≥ 1.5 and *q*-value < 0.05.

**Figure 3:**
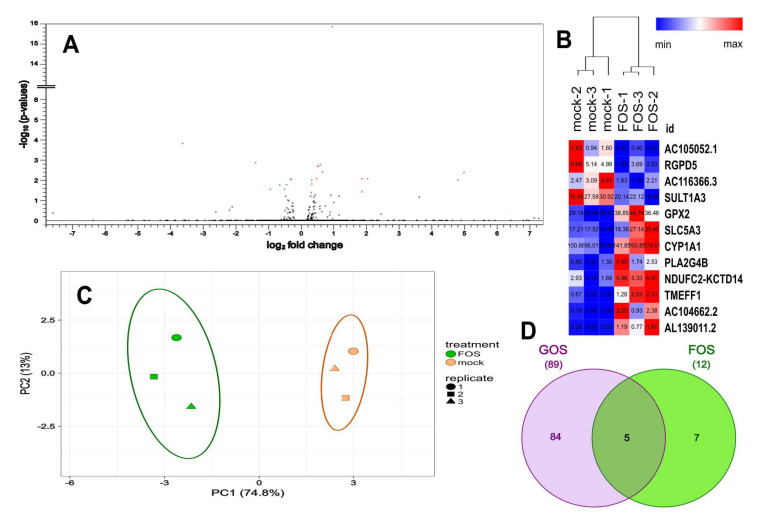
Differentially expressed genes associated with FOS treatment. (**A**) Volcano plot of genes differentially expressed between FOS and FOS mock treatment (*n* = 3). The abscissa represents log_2_ fold changes in gene expression, the ordinate represents statistical significance of the variations in gene expression, expressed as -log_10_ (*p*-values). (**B**) Principal component scatter plot showing variance of the biological replicates for each treatment (*n* = 3). Axis x and y show principal components 1 (PC1) and 2 (PC2) that explain 74.8% and 13% of the total variance, respectively. Prediction ellipses indicate probability 0.95 that a new observation from the same group will fall inside the ellipse; N = 6 data points. (**C**) Heatmap displaying differentially expressed genes for FOS and mock treatments. Rows and columns are clustered using Pearson’s correlation distance and average linkage. Data are presented as the normalized transcripts count (TPM) with |FC| ≥ 1.5 and *q*-value < 0.05. (**D**) Venn diagram showing the overlap of differentially expressed genes between GOS and FOS treatments. Data are presented as the number of genes with |FC| ≥ 1.5 and *q*–value < 0.05.

Volcano plots were generated to visualize the distribution of differentially expressed genes following GOS and FOS exposure (Figure 2A and Figure 3A respectively), which showed a limited number of genes modulated by FOS when compared to GOS. Principal component analysis of the normalized transcript counts (TPM) disclosed a tight relationship between biological replicates within each treatment group such that oligosaccharides-exposed samples clustered distinctly from their mock-treated counterparts. As a result, the first two principal components taken together explained 91.9% and 87.8% of the variability among the samples exposed to GOS and FOS when compared to mock treated cells (Figure 2B and Figure 3B respectively). We further examined the distribution of differentially expressed genes exhibiting FDR *p*-adj < 0.05 and |FC| ≥ 1.5 using heatmaps, which are presented in Figure 2C and Figure 3C for the GOS and FOS treatments respectively. Following exposure to GOS, 89 genes were differentially expressed according to the criteria, of which 53 were up-regulated and 36 down-regulated when compared to control mock-treated cells (Figure 2C and Table S1). Whereas for FOS, the total number of genes fulfilling the criteria for differential expression was limited to 12, comprising of eight up-regulated and four down-regulated genes (Figure 3C and Table S1). GOS- and FOS-treated cells shared five differentially expressed genes as presented in the Venn diagram shown in Figure 3D: *TMEFF1*, *GPX2*, *SLC5A3*, *SULT1A3*, *AL139011*.*2*. Three of these genes were up-regulated upon exposure to GOS and FOS treatments (*GPX2*, *SLC5A3* and *AL139011.2*) and two genes showed the opposite modulation such that *TMEFF1* transcripts were reduced upon GOS exposure whilst increased with FOS treatment, and *SULT1A3* transcription was increased with GOS but reduced with FOS exposure.

### 3.4. Validation of Differentially Expressed Genes by RT-qPCR

**Figure 4:**
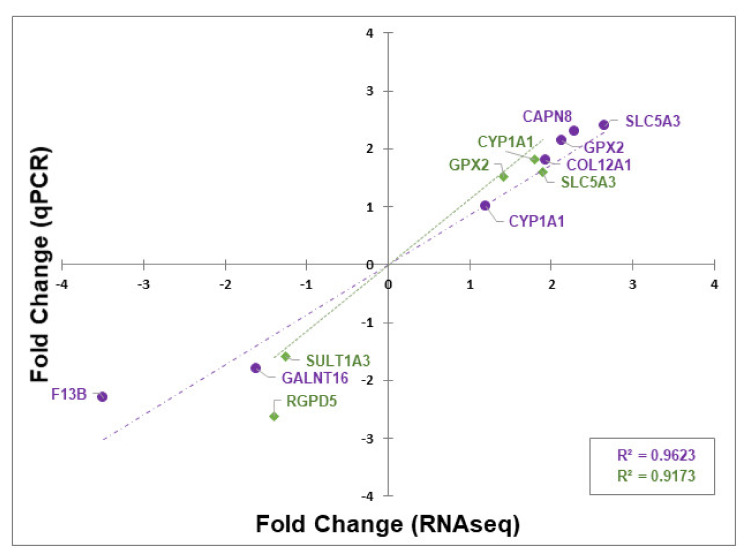
Validation of RNA-seq by RT-qPCR. The abscissa represents RT-qPCR Fold Change in gene expression, the ordinate represents RNA-seq Fold Change in gene expression. ● GOS gene expression; −∙−∙ GOS gene expression linear trend line; ♦ FOS gene expression; …. FOS gene expression linear trend line.

RNA-seq data was independently validated by RT-qPCR measurement of the transcript levels of genes identified as differentially expressed (RT-qPCR raw data presented in Table S2). Results revealed strong correlation between the RNA-seq and RT-qPCR differential expression data for the GOS and FOS treatments (Figure 4; GOS R^2^ = 0.9623 and FOS R^2^ = 0.9173). Accordingly, genes *GPX2* (RT-qPCR GOS FC = +2.1; FOS FC = +1.4) and *SLC5A3* (RT-qPCR GOS FC = +2.7; FOS FC = +1.9) were found up-regulated simultaneously with GOS and FOS treatments. Consistent with the GOS RNA-seq data, genes *F13B* (RT-qPCR FC = −3.6) and *GALNT16* (RT-qPCR FC = −1.6) were found to be down-regulated whilst *COL12A1* (RT-qPCR FC = +1.9) and *CAPN8* (RT-qPCR FC = +2.3) were up-regulated. Similarly, congruency was observed for the FOS treatment-associated up-regulated gene *CYP1A1* (RT-qPCR FC = +1.7) and down-regulated genes *SULT1A3* (RT-qPCR FC = −1.3) and *RGPD5* (RT-qPCR FC = −1.4).

### 3.5. Functional Analysis of Differentially Expressed Genes

Biological attribute identification and functional interpretation of our data was achieved by implementing bioinformatics tools on gene set enrichment and pathway database analyses based on Gene Ontology (GO) terms and the Kyoto Encyclopedia of Genes and Genomes (KEGG) collection of databases. The KEGG knowledge base links gene catalogues to functional information such as metabolism, membrane transport, signal transduction and cell cycle in pathway assemblies. GO resource provides computable knowledge for biological functions of genes and gene products structured within biological process, molecular function and cellular component classes.

**Figure 5:**
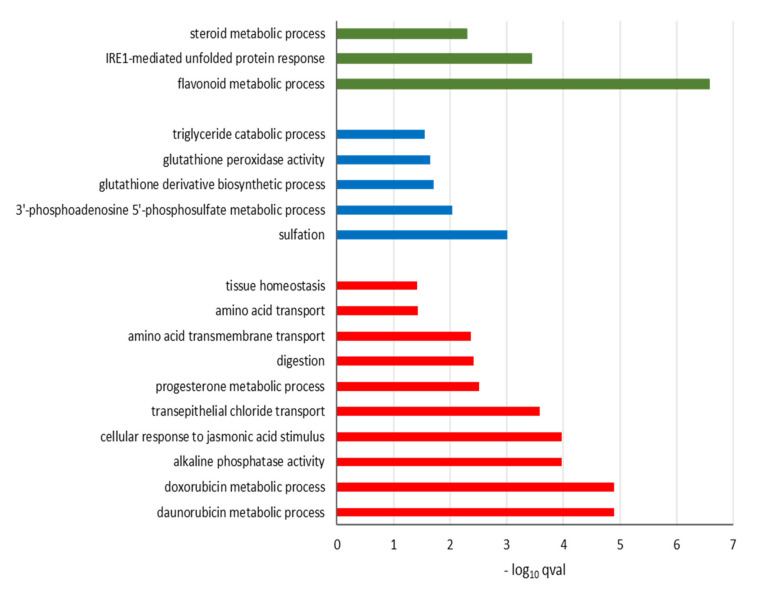
Gene Ontology (GO) enrichment analysis of differentially expressed genes of oligosaccharides treated compared to mock treated Caco–2 cells. Differentially expressed genes (FDR *p*–adj. < 0.05, |FC| ≥ 1.5) were clustered according to GO bioprocesses classification. ■ GOS up-regulated; ■ GOS down-regulated; ■ FOS up-regulated.

GO classification of the 89 differentially expressed (DE) genes associated with GOS exposure placed the up-regulated genes within 10 GO terms (Figure 5) describing metabolic processes (daunorubicin, doxorubicin and progesterone metabolic processes, alkaline phosphatase activity and digestion), cell membrane transport (transepithelial chloride transport, amino acid transmembrane transport) and tissue homeostasis. KEGG analysis of the gene sets (Table 5) indicated the up-regulated gene-associated pathways were enriched for metabolic processes featuring folate biosynthesis (hsa00790), thiamine metabolism (hsa00730), thyroid hormone synthesis (hsa04918), protein digestion and absorption (hsa04974), membrane transport with salivary (hsa04970) and pancreatic (hsa04972) secretion. The GOS down-regulated genes were placed in five GO terms (Figure 5) that identified processes related to sulfation, 3’-phosphoadenosine-5’-phosphosulfate sulfotransferase, glutathione derivative biosynthesis, glutathione peroxidase activity and triglyceride catabolism. The down-regulated GO terms were further identified in KEGG (Table 5) as reduced glutathione metabolism (hsa00480), chemical carcinogenesis (hsa05204), drug and xenobiotics metabolism-cytochrome P450 (hsa00982 and hsa00980). A further seven DE genes were enriched in a unique GO cell compartment described as the collagen-containing extracellular matrix (GO:0062023, *p*–adj.= 0.018) involving three down-regulated and four up-regulated genes (Table 6).

Gene Ontology analysis of the 12 DE genes linked with FOS treatment revealed bioprocesses (Figure 5) involved in flavonoid metabolism, IRE1-mediated unfolded protein response and steroid metabolism represented by the genes *SULT1A3* (FC = –1.6, *p*–adj. = 0.0178), *CYP1A1* (FC = +1.8, *p*-adj. < 1.10^−12^ ) and *PLA2G4B* (FC = +3.7, *p*–adj. = 0.008). Analysis of the KEGG pathways (Table 5) revealed increases in pathways linked with ovarian steroidogenesis (hsa04913), arachidonic acid metabolism (hsa00590) and general metabolic pathways (hsa01100). GO analysis also identified the mitochondrial inner membrane (GO:0005743) as a unique cell compartment, as represented by three up-regulated genes *CYP1A1*, *PLA2G4B* and *NDUFC2*-*KCTD14* (*p*–adj. = 0.002, Table 6).

## 4. Discussion

Non-digestible oligosaccharides are prebiotics able to selectively stimulate the components of gut microbiota beneficial to host health [47]. A consequence of non-digestion is that they will reach the colon intact to exert any non-prebiotic effects on the intestinal epithelium. In this study, we addressed the hypothesis that oligosaccharides could directly affect the transcriptome of epithelial cells representative of the intestinal mucosa and have exploited RNA-seq methodology to describe the transcriptome of confluent Caco–2 cell monolayers exposed to the GOS and FOS.

The Caco–2 cell line model was chosen for its ability to form tight junctions and microvilli, thus expressing enzymes and transporters characteristic of enterocytes [48,49]. A number of in vitro studies have evaluated the impact of various prebiotics on the cell barrier integrity when subject to pathogen-induced barrier disruptions [50,51], inflammation-inducing cytokines and lipopolysaccharides [52] or toxin-induced challenge with the mycotoxin deoxynivalenol [53]. Here, exposure to GOS (1.4% w/v) increased trans-epithelial electrical resistance of the monolayer suggesting GOS can improve the integrity of the tights junctions. FOS (2% w/v) exposed cells similarly increased trans-epithelial electrical resistance despite marginally failing to meet significance. We sought to understand these effects and any wider transcriptional responses to GOS and FOS by establishing differential gene expression using RNA-seq. Differential expression was determined in prebiotic-treated Caco–2 cell monolayers with respect to tailored mock exposure to the saccharides present in the GOS and FOS preparations.

**Figure 6:**
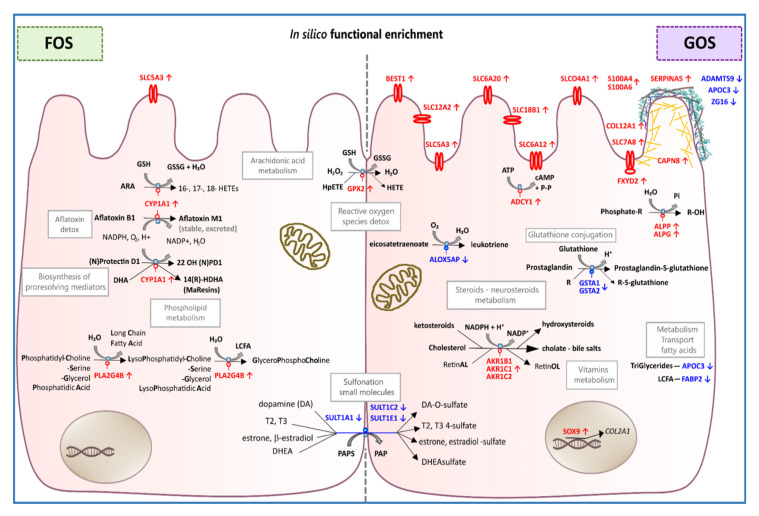
Summary of the pathway associations of the differentially expressed genes (FDR p-adj. < 0.05, |FC| ≥ 1.5) identified as responding to the prebiotic oligosaccharides FOS or GOS in confluent colonic Caco–2 cells. Pathways were deduced from GO enrichment and KEGG analyses. Up-regulated functions are marked in red text and the down-regulated, in blue text.

Differentially expressed genes were used as the basis for in silico approaches to assess the functional consequences. The biological significance of a given fold-change is likely to depend on the gene and on the experimental context, and for this reason, universal thresholds are not applied for the bioinformatic interrogation of transcriptomic data [54,55,56]. We have adopted a stringent statistical approach through the use of Benjamini and Hochberg’s false discovery rate correction for multiple testing to establish the criteria of FDR adjusted probability *p*-adj < 0.05 and an inclusive absolute fold change ≥ 1.5 to identify differential gene expression. We opted for the inclusive value of ≥ 1.5-fold change since it has been demonstrated that the enrichment of biologically relevant functions can occur at low fold changes in RNA levels when appropriate statistical methods are employed [57]. However, despite steady-state observations that protein levels are generally determined by transcript concentrations, there are frequent differences observed between transcriptomic and proteomic assessments of expression during cellular transitions [58]. A total of 89 DE genes were identified between GOS and mock treated cells, whereas for FOS exposure the number was reduced to 12 DE genes. The RNA-seq data from both experiments were validated by RT-qPCR using specific primer sets. Strong correlations were observed between the fold-change determined for the two methods with R^2^ = 0.96 for GOS and R^2^ = 0.92 for FOS treatments. The DE data were used for GO term enrichment and KEGG analyses to identify transcriptional pathways responding to prebiotic oligosaccharide exposure. Modulation of the pathways identified are discussed below and are summarized in Figure 6 for reference.

### 4.1. FOS Treatment Associated with 12 Differentially Expressed Genes

FOS treatment resulted in 12 DE genes comprising of four down-regulated and eight up-regulated genes. GO terms identified the up-regulated genes *SULT1A3*, *CYP1A1* and *PLA2G4B* as related to flavonoid metabolism, IRE1-mediated unfolded protein response and steroid metabolism. Interrogation of the KEGG database confirmed these three genes are functional in ovarian steroidogenesis and arachidonic acid metabolism (FDR *q*–val < 0.001) and general metabolic pathways (FDR q–val = 0.013). *CYP1A1* encodes a Cytochrome P450 monooxygenase exhibiting high catalytic activity for steroid hormones [59] and hydroxylation of certain polyunsaturated fatty acids (PUFA) [60], whereas *PLA2G4B* encodes phospholipase A2 that preferentially catalyzes hydrolysis of arachidonoyl phospholipids to release lysophospholipids and fatty acids, with a preference for arachidonoyl phospholipids. The products of these genes also form a nexus with the phosphatidylinositol 4-kinases products of *AC104662.2* and *PI4K2B* around cell signaling, lipid and membrane trafficking pathways [61]. In the absence of direct evidence for any transcriptional changes of the genes encoding tight junction proteins to affect the increases in TEER observed, it is conceivable that FOS exposure results in compositional changes in membrane sterols and phospholipids that affect the physical properties of the cell membrane, altering rigidity/fluidity leading to stabilization or disruption [62]. This contrasts with the direct effect, although not attributed to oligosaccharides, reported on the expression of *CLDN4*, encoding the epithelial cell tight junction protein Claudin 4, in Caco–2 cells exposed to complex food homogenates of apple, broccoli and button mushroom [63].

### 4.2. GOS Treatment Associated with 53 up-Regulated Genes

GOS treatment led to 53 up-regulated genes that associated with 10 GO terms. Genes *AKR1C1*, *AKR1C2* and *AKR1B1* were related to the terms daunorubicin and doxorubicin metabolic process, cellular response to jasmonic acid stimulus, progesterone metabolic process and digestion (FDR q-val < 0.005). These genes encode enzymes harboring oxidoreductase and aldo-keto reductase activities that target steroids and prostaglandins with varying levels of bile acid-binding affinity. Progesterone metabolic process and cellular response to jasmonic acid stimulus relate also to up-regulation of *AKR1C1* and *AKR1C2* genes due to their catalytic activity related to androgens inactivation (progesterone and testosterone respectively). Despite its plant origin, jasmonic acid is an oxylipin-signaling molecule often considered a structural and functional analogue to prostaglandins in animals. Mammalian eicosanoids and oxylipins such as prostaglandins and leukotrienes are active signaling molecules derived from oxygenated PUFAs and are potent regulators of host immune responses. Notably, mammalian prostaglandins and leukotrienes (products of arachidonic acid metabolism) can polarize macrophages, modulate T helper cell immune responses, stimulate chemokine production, phagocytosis, lymphocyte proliferation and leukocyte chemotaxis [64].

GO also associated *AKR1C1* and *AKR1C2* with *CAPN8* within digestion processes. *CAPN8* encodes tissue-specific calpain 8a (cytosolic calcium-dependent cysteine protease), which in mice is reported to be expressed specifically in the mucus-secreting pit cells of the gastric mucosa as well as in a subset of goblet cells in the small intestine [65]. Calpains are also implicated in membrane trafficking processes due to their localization in endoplasmic reticulum, Golgi and lipid rafts [66,67]. The KEGG protein digestion and absorption pathway (hsa04974) identified the genes *SLC7A8*, *COL12A1* and *FXYD2*, which respectively encode L-type amino acid transporter 2, collagen type XII alpha 1 chain and Na/K-transporting ATPase subunit gamma suggesting specific amino acid transport and focused structural protein remodeling. The KEGG pancreatic secretion (hsa04972) and salivary secretion (hsa04970) pathways featured the up-regulated genes *SLC12A2* encoding a basolateral Na-K-Cl symporter and *ADCY1* encoding Ca^2+^/calmodulin-activated adenylyl cyclase. These activities are linked as Ca^2+^ and cAMP can regulate intestinal Na-K-Cl symporter activity [68]. Related to this, the GO terms transepithelial chloride transport (FDR q-val < 0.001) and amino acid transport (FDR q-val < 0.05) identified genes for ion channel and solute carrier proteins (*BEST1*, *SLC12A2*, *SLC6A20*, *SLC7A8* and *SLC6A12*). Several solute carrier encoding genes were up-regulated (*SLC7A8, SLC6A20, SLC6A12, SLCO4A1, SLC12A2, SLC5A3* and *BEST1*) that feature in Reactome pathway analyses related to amino acid transport across the plasma membrane and the transport of small molecules. Two bioprocesses relevant to the digestion pathway were enriched in KEGG folate biosynthesis (hsa00790) and thiamine metabolism (hsa00730) pathways. Genes *ALPP* and *ALPG* encoding intestinal and placental-like alkaline phosphatase respectively were identified with *AKR1B1* encoding aldo-keto reductase. *AKR1B1* in association with *SOX9* was identified as enriched constituents of the GO tissue homeostasis pathway. *SOX9* encodes SRY-Box Transcription Factor 9 that binds to the *COL2A1* promoter to activate cartilaginous tissue-specific Collagen Type II Alpha 1 Chain protein expression, which has an essential role in skeletal development and contributes to the ability of cartilage to resist compressive forces. Collectively, these data are consistent with the hypothesis that GOS elicits energy-dependent transmembrane trafficking of solutes in Caco–2 cell monolayers accompanied by collagen and cytoplasmic membrane remodeling that may be linked to the observed increases in TEER.

### 4.3. GOS Treatment Associated with 36 Down-Regulated Genes

Of 36 DE genes found down-regulated following GOS exposure, seven DE genes were annotated in enriched GO bioprocess terms associated with sulfation and 3’-phosphoadenosine 5’-phosphosulfate metabolic process (genes *SULT1E1* and *SULT1C2*), glutathione peroxidase activity and derivative biosynthetic process (genes *GSTA2*, *GSTA1* and *ALOX5AP*), and triglyceride catabolic process (genes *APOC3* and *FABP2*). All these genes encode proteins related to fatty acid and arachidonic acid metabolism. Genes *SULT1E1* and *SULT1C2*, encode sulfotransferases responsible for sulphate conjugation of many hormones (such as estrogens), neurotransmitters and xenobiotic compounds. *GSTA2* and *GSTA1* encode alpha-glutathione S-transferases (GSTs) involved in the synthesis of prostaglandins (PGs) and leukotrienes and are thus responsible for the metabolization of electrophilic compounds including carcinogens, therapeutic drugs and the by-products of oxidative stress. Notably, through eicosanoid metabolites GSTs can play a central role as regulators of transcription factor peroxisome proliferator-activated receptor γ (PPARγ) [69,70]. In mammals, overexpression of GSTs in tumor cells has been implicated with resistance to various anti-cancer agents and chemical carcinogens and in microbes, plants and mammals, expression of GSTs are up-regulated by exposure to pro-oxidants, thus suggesting that induction of GST is an evolutionary conserved response of cells to oxidative stress [71]. *ALOX5AP* encodes arachidonate 5-lipoxygenase activating protein required for leukotriene biosynthesis by 5-lipoxygenase (ALOX5). The products of LOX metabolism either represent biologically active eicosanoid metabolites such as hydroperoxy–eicosatetraenoic acids (HPETEs) or give rise to the production of reactive oxygen species (ROS). A functional role has also been attributed to lipoxygenase-catalyzed arachidonic and linoleic acid metabolism in cancer development [72,73]. *APOC3* encodes apolipoprotein C3, a protein component of the triglyceride (TG)-rich lipoproteins (TRLs) and FABP2 encodes intestinal-type Fatty Acid-Binding Protein—an intracellular fatty acid-binding protein that participates in the uptake, metabolism and transport of long-chain fatty acids. This lends further support to the reconfiguration of fatty acid metabolism in response to GOS treatment whilst maintaining homeostasis.

GO cell compartment analysis identified the collagen-containing extracellular matrix based on the down-regulation of *APOC3*, *ZG16* and *ADAMTS9* and up-regulation of *COL12A1*, *S100A4*, *S100A6* and *SERPINA5*. Gene *APOC3* encodes a liver and small intestine-secreted apolipoprotein C-III (apoC-III) found with triglyceride-rich lipoproteins, such that increases in apoC-III levels induces the development of hypertriglyceridemia and overexpression can contribute to coronary heart disease in humans [74,75]. Following exposure to GOS, *APOC3* was down-regulated thus suggesting that a reduction in apoC-III levels could be beneficial for triglyceridemia. However, *ZG16* and *ADAMTS9*, which have been ascribed roles in the control of cancer development, were also down-regulated. Zymogen granule protein 16 (ZG16) is one of the most significantly down-regulated genes in colorectal cancer (CRC) and harbors a jacalin-like lectin domain. Lectins are carbohydrate-binding proteins able to detect subtle differences between complex carbohydrate structures, thus recognizing specific sugars to carry out various functions including cell attachment, migration and invasion. Amongst these, some galectins share an affinity for simple β-galactoside moieties [76]. ZG16 may be an important component of the protective mucus layer, which helps separate host epithelium from commensal bacteria in the colon [77]. The high similarity of ZG16 to jacalin suggests that the human homologue may play an important role in colon cancer immunity. The loss of ZG16 associated with CRC development has led to the hypothesis that ZG16 reduction may disrupt well-organized bacterial surveillance systems to facilitate bacterial invasion of host tissues and cause local inflammatory changes, which may constitute an increased risk for the development of cancer [78]. *ADAMTS9* encodes a member of the ADAMTS (a disintegrin and metalloproteinase with thrombospondin motifs) protein family, a secreted mammalian metalloprotease that localizes to the cell-surface and/or extracellular matrix. The extracellular matrix is continuously remodeled by coordinated biosynthesis and proteolysis of its components. Active proteolytic ADAMTS9 has been shown to interact with fibronectin and disrupt fibril networks [79]. Furthermore, it has been suggested ADAMTS9 contributes to the suppression of tumorigenesis by decreasing cell proliferation, inducing cell apoptosis and inhibiting angiogenesis through regulating the AKT/mTOR signaling pathway, whilst methylation of *ADAMTS9* genes is associated with poor survival of gastric cancer patients [80].

Collagen-containing extracellular matrix enriched genes *S100A4* and *S100A6* were found to be up-regulated following treatment with GOS. Proteins S100A4 and S100A6 are members of the S100 calcium binding protein family that exert biological functions via interactions with target proteins. Many kinds of cells including fibroblasts, immune cells, and cancer cells can produce S100A4, which are released into the extracellular space in response to various stimuli such as activated normal T cell expression and secreted factors (RANTES) produced by the tumor cells. S100A4 could be involved in the regulation of cell proliferation and differentiation, apoptosis, Ca^2+^ homeostasis, and energy metabolism [81]. Sun et al. demonstrated that mice deficient in S100A4 exhibited impaired humoral and cellular immunity after mucosal immunization using Cholera toxin as adjuvant, revealing a reduced production of Th1, Th2 and Th17 cytokines [82]. Protein S100A6 has been described in a limited number of cell types in adult normal tissues and in several tumor cell types. As an intracellular protein, S100A6 has been implicated in the regulation of several cellular functions, such as proliferation, apoptosis, cytoskeleton dynamics and cellular response to different stress factors. S100A6 can be secreted/released by certain cell types which points to extracellular effects of the protein such as antimicrobial activity [83]. Similar up-regulation was observed for gene *SERPINA5* encoding Protein C inhibitor (PCI), a serine protease inhibitor of serpin type that is found in most tissues and fluids, including blood plasma, seminal plasma and the urine of humans [84]. As an antimicrobial agent, PCI has the ability to disrupt the bacterial cell wall to cause death by interacting with lipid membranes leading to permeabilization of bacterial pathogens [85]. PCI also inhibits proteases of the blood coagulation and fibrinolysis system, whilst in cancer cells it suppresses tumor invasion by inhibiting urokinase-type plasminogen activators and inhibits tumor growth and metastasis, which are independent of its protease-inhibitory activity [86]. Lastly, *COL12A1* encoded collagen XII is a member of the family of fibril-associated collagens with an interrupted triple helix (FACIT) structure. Mutations in the collagen XII gene associated with extracellular matrix-related myopathy, as the protein functions to preserve muscle and bone architecture through collagen fibril organization [87,88].

## 5. Conclusions

In this study, the direct effects of GOS and FOS on colonic epithelial cells were assessed through changes in monolayer transepithelial resistance and transcriptome analysis. Results suggest GOS have the potential to directly increase integrity of the epithelial barrier. Transcriptome data suggest TEER increased independent of specific tight junction protein expression but could be due to sterol/fatty acid compositional changes associated with transporter-dependent remodeling of the cell membrane. Specific candidate pathways group the genes involved in digestion and transepithelial transport, which contribute to intestinal cell integrity and function.

Carbohydrates have enormous potential to encode biological information. It is conceivable that GOS and FOS can crosstalk with cells using lectin molecules as the interface. Typically, lectins and their complimentary carbohydrate are located on the surfaces of opposing cells, which can be of the same type or different types. Such interactions are required for cell differentiation, development and pathological states. These results highlight concerted effects on transmembrane trafficking, differences in xenobiotic biotransformation, and the production of antimicrobial agents.

GOS and FOS treatments shared relatively few differentially expressed genes, suggesting they have different modes of action. Our strategy has produced a comprehensive database of gene expression profiles of Caco–2 cell monolayers exposed to oligosaccharide food ingredients, allowing further work to link gene expression signatures of cultured cells to their mode of action, and thus potentially facilitating product choice in human or animal intervention studies.

## Figures and Tables

**Table 1:** Nutrabiotic^®^ galacto-oligosaccharides (GOS) and Orafti^®^L95 fructo-oligosaccharides (FOS) syrup composition.

	Composition
**Nutrabiotic^®^ GOS**	75% (w/w) dry solids (ds)
Galacto-oligosaccharides	66.5 (w/w ds)
Lactose	10.1 (w/w ds)
Glucose	21.8 (w/w ds)
Galactose	1.6 (w/w ds)
**Orafti^®^ L95 FOS**	74.7% (w/w) dry solids
Fructo-oligosaccharides	94.8 (w/w ds)
Fructose	3 (w/w ds)
Sucrose	2 (w/w ds)
Glucose	0.2 (w/w ds)

**Table 2:** Culture media composition.

Media	Basic media* supplementation
**Cell growth & confluence**	
	+ 10% (v/v) FBS
	+ antibiotics 100 U/mL (penicillin, streptomycin)
**Conditioning & treatment**	
GOS 2%	+ 2% (v/v) Nutrabiotic^®^ GOS
FOS 2%	+ 2% (v/v) Orafti^®^ L95 FOS
GOS mock	+ 0.67 % (m/m) mono- and di-saccharides (glucose 4.796 g/L, galactose 0.352 g/L, lactose 2.126 g/L)
FOS mock	+ 0.104 % (m/m) mono- and di-saccharides (glucose 0.044 g/L, fructose 0.660 g/L, sucrose 0.421 g/L)

* basic media: Dulbecco’s MEM medium (DMEM), L-glutamine 2mM, MEM non-essential amino acids supplement 1x, Fetal Bovine Serum (FBS).

**Table 3:** Primer sequences for RNA-seq validation by qPCR.

Target Gene	Primer Sequence (5’–3’)	Product Size (bp)	NCBI Accession	RNA–Seq Identifier
				
CAPN8	F: GGTCTAGGTGACTGCTGGCT R: AGCAGCTGTCCATTCTTGGT	197	NM_001143962.2	ENSG00000203697
COL12A1	F: GGCAAGGCTATCCAGGTTCC R: TAAGCACGTGCGCAAACATC	106	NM_004370.6	ENSG00000111799
CYP1A1	F: CCCCCACAGCACAACAAGAG R: GGGTGAGAAACCGTTCAGGT	146	NM_000499.5	ENSG00000140465
F13B	F: GGACACTTCCTCCTGAGTGTGT R: CGTCTGCAACAGCCCCATTC	81	NM_001994.3	ENSG00000143278
GALNT16	F: CTGACCTTCGTGGAGGGTTC R: GGTCTGTCCGGGTCATCTTC	84	NM_020692.3	ENSG00000100626
GPX2	F: TTTCAATACGTTCCGGGGCA R: CTGACAGTTCTCCTGATGTCCA	169	NM_002083	ENSG00000176153
RGPD5	F: CAAGAAATTGCCTGTGCCCC R: TCCATCGAGGTGGTGTTTCG	215	NM_005054.3	ENSG00000015568
SLC5A3	F: ATGCAGCGGGGTTGGTACA R: AGCAACACAGCAGGGTCAAA	235	NM_006933.7	ENSG00000198743
SULT1A3	F: CGGTCTCCTACTACCATTTCC R: AGGACCCGTAGGACACTTC	108	NM_177552.3	ENSG00000261052
ACTB	F: CTGGAACGGTGAAGGTGACA R: AAGGGACTTCCTGTAACAATGCA	140	NM_001101.5	
GAPDH	F: GGAGTCCACTGGCGTCTTCAC R: GAGGCATTGCTGATGATCTTGAGG	165	NM_002046.7	
PUM1	F: TGAGGTGTGCACCATGAAC R: CAGAATGTGCTTGCCATAGG	187	NM_014676.2	

**Table 4:** Summary of sequencing reads mapped to the human reference genome. RNA sequencing was strand-specific and only reverse strand reads were mapped to the reference genome Homo sapiens Hg38. FOS (1–3) and GOS (1–3) are oligosaccharide-treated groups, the experiment specific control treatment groups are referred to as “mock” (1–3); each treatment consists of three independent biological replicates (labelled 1–3) that were created from a pool of three technical replicates (*n* = 3).

		Raw Read Count	Ignored Reads (Wrong Strand)	Reads Paired and Mapped	Fragments Mapped to Genes	Fragments Mapped as Intergenic	Protein Coding Genes
**FOS experiment**	**mock1**	47,141,326	615,415 (1.30%)	88.25%	95.87%	4.13%	96.47%
	**mock2**	49,710,422	771,068 (1.55%)	87.24%	95.81%	4.19%	96.54%
	**mock3**	44,983,458	717,544 (1.59%)	89.62%	95.86%	4.14%	96.64%
	**FOS1**	49,264,466	842,951 (1.71%)	85.56%	95.47%	4.53%	96.35%
	**FOS2**	51,905,580	749,101 (1.44%)	86.80%	95.64%	4.36%	96.46%
	**FOS3**	49,519,788	688,065 (1.39%)	89.41%	95.46%	4.54%	96.59%
**GOS experiment**	**mock1**	62,474,912	819,911 (1.31%)	89.96%	95.73%	4.27%	96.47%
	**mock2**	45,810,896	589,565 (1.29%)	91.01%	95.86%	4.14%	96.59%
	**mock3**	49,459,368	649,851 (1.31%)	88.43%	95.88%	4.12%	96.48%
	**GOS1**	134,054,024	2,460,936 (1.83%)	89.29%	95.78%	4.22%	96.54%
	**GOS2**	59,524,110	1,153,619 (1.94%)	87.68%	95.87%	4.13%	96.49%
	**GOS3**	56,118,898	809,112 (1.44%)	90.74%	95.53%	4.47%	96.61%

**Table 5:** Kyoto Encyclopedia of Genes and Genomes (KEGG) pathway analysis. Enriched pathways among the differentially expressed genes were identified by KEGG analysis. Differentially expressed gene false discovery rate (FDR) p-adj.< 0.05 and absolute value of fold change ≥1.5.

KEGG Pathway	FDR *q*.val	Enriched Gene	Protein Coding Alias	Fold Change (mRNAseq)	FDR *p*–Value (mRNAseq)
**GOS (UP)**					
hsa00790 Folate biosynthesis	0.0003	*AKR1B1*	Aldo-Keto Reductase	1.8	1 × 10^−11^
		*ALPP*	Intestinal Alkaline Phosphatase	2.1	4 × 10^−10^
		*ALPG*	Placental-Like Alkaline Phosphatase	2.1	2 × 10^−7^
hsa00730 Thiamine metabolism	0.0025	*ALPP*			
		*ALPG*			
hsa04918 Thyroid hormone synthesis	0.0170	*GPX2*	Glutathione Peroxidase-Gastrointestinal	2.1	<1 × 10^−12^
		*ADCY1*	Ca^2+^/Calmodulin-Activated Adenylyl Cyclase	1.5	5 × 10^−4^
		*FXYD2*	Sodium/Potassium-Transporting ATPase Subunit Gamma	1.8	0.010
hsa04970 Salivary secretion	0.0359	*SLC12A2*	Basolateral Na-K-Cl Symporter	1.5	0.004
		*ADCY1*			
		*FXYD2*			
hsa04974 Protein digestion and absorption	0.0359	*SLC7A8*	L-Type Amino Acid Transporter 2	1.6	9 × 10^−7^
		*COL12A1*	Collagen Type XII Alpha 1 Chain	1.8	2 × 10^−12^
		*FXYD2*			
hsa04972 Pancreatic secretion	0.0495	*SLC12A2*			
		*ADCY1*			
		*FXYD2*			
**GOS (DOWN)**					
hsa00480 Glutathione metabolism	0.002	*ODC1*	Ornithine Decarboxylase 1	−1.5	1 × 10^−11^
		*GSTA2*	Glutathione S-Transferase Alpha 2	−1.5	4 × 10^−5^
		*GSTA1*	Glutathione S-Transferase Alpha 1	−1.5	0.008
hsa00982 Drug metabolism - cytochrome P	0.005	*GSTA2*			
hsa00980 Metabolism of xenobiotics by cytochrome P	0.007	*GSTA1*			
hsa05204 Chemical carcinogenesis	0.009	*ADH6*	Alcohol Dehydrogenase 6 (Class V)	−1.7	0.004
**FOS (UP)**					
hsa04913 Ovarian steroidogenesis	0.0001	*CYP1A1*	Cytochrome P450 Family 1 Subfamily A Member 1 (Aryl Hydrocarbon Hydroxylase)	1.8	<1 × 10^−12^
		*PLA2G4B*	Phospholipase A2 Group IVB	3.7	0.008
hsa00590 Arachidonic acid metabolism	0.0002	*PLA2G4B*			
		*GPX2*	Gastrointestinal Glutathione Peroxidase	1.5	0.002
hsa01100 Metabolic pathways	0.0130	*NDUFC2-KCTD14*	NDUFC2-KCTD14 Readthrough Transcript Protein (NADH Dehydrogenase (Ubiquinone) 1 Subunit C2, Isoform 2)	3.7	0.039
		*AC104662.2*	predicted type II PI4 kinase protein family (PI4K2B)	28.2	0.009
		*PLA2G4B*			
		*CYP1A1*			

**Table 6:** Cell compartment GO enrichment analysis of differentially expressed genes.

GO Cell Compartment Enrichment	FDR q.val	Enriched Gene	Protein Coding Alias	Fold Change (mRNAseq)	FDR *p*–Value (mRNAseq )
**GOS**					
GO:0062023 Collagen-containing extracellular matrix	0.018	*ZG16*	Zymogen Granule Protein 16 (Jacalin-Like Lectin Domain Containing)	−1.6	9 × 10^−7^
		*APOC3*	Apolipoprotein C3	−1.5	3 × 10^−5^
		*ADAMTS9*	A Disintegrin And Metalloproteinase with ThromboSpondin Motifs 9	−1.5	4 × 10^−2^
		*SERPINA5*	Serine (Or Cysteine) Proteinase Inhibitor, clade A (Alpha-1 Antiproteinase, Antitrypsin), Member 5	2.1	<1 × 10^−12^
		*COL12A1*	Collagen type XII Proteoglycan	1.8	2 × 10^−12^
		*S100A4*	S100 Calcium Binding Protein A4	1.7	2 × 10^−10^
		*S100A6*	S100 Calcium-Binding Protein A6 (Calcyclin)	1.5	5 × 10^−5^
**FOS**					
GO:0005743 Mitochondrial inner membrane	0.002	*CYP1A1*	Cytochrome P450 Family 1 Subfamily A Member 1 (Aryl Hydrocarbon Hydroxylase)	1.8	<1 × 10^−12^
		*PLA2G4B*	Phospholipase A2 Group IVB	3.7	0.008
		*NDUFC2-KCTD14*	NDUFC2-KCTD14 Readthrough Transcript Protein (NADH Dehydrogenase (Ubiquinone) 1 Subunit C2, Isoform 2)	3.7	0.039

## Supplementary Materials

The following are available online at https://www.mdpi.com/2072-6643/12/5/1281/s1, Supplementary Table S1: GOS and FOS gene expression browser, Table S2: RT-qPCR raw data.
